# Ikaros Deletions among Bulgarian Patients with Acute Lymphoblastic Leukemia/Lymphoma

**DOI:** 10.3390/diagnostics14171953

**Published:** 2024-09-03

**Authors:** Stefan Lozenov, Yoanna Tsoneva, Georgi Nikolaev, Rossitza Konakchieva

**Affiliations:** 1 Specialized Hospital for Active Treatment of Hematology Diseases, 1756 Sofia, Bulgaria; 2 Department of Cell and Developmental Biology, Faculty of Biology, Sofia University “St. Kliment Ohridski”, 1164 Sofia, Bulgaria

**Keywords:** *Ikaros*, breakpoint-specific qPCR, acute lymphoblastic leukemia

## Abstract

The *Ikaros* zinc finger factor 1 is a transcription factor with a well-known role in B- and T-cell development. The deletions of *IKZF1* have an established significance in acute lymphoblastic leukemia, while reports on its prevalence and prognostic significance among ALL subtypes and regions vary. Breakpoint-specific qPCR is a practical method for testing of the most frequent types of *IKZF1* deletions, considering there is clustering of the deletion events. The most commonly reported deletions are Δ4–7, Δ4–8, Δ2–7, and Δ2–8, with deletion Δ4–7 being the most common one. We retrospectively administered a breakpoint-specific qPCR design for screening for the most frequent types of *IKZF1* deletions to 78 ALL patients that were diagnosed and treated between 2010 and 2022. We observed the products through gel electrophoresis, and we conducted descriptive statistics, EFS, and OS analyses. Our study found 19 patients with *IKZF1* deletions, with two subjects manifesting more than one deletion. The prevalence in the different subgroups was as follows: Ph/+/ B-ALL 46%, Ph/−/ B-ALL 30%, T-ALL/LBL 4%. There was a statistically significant difference in EFS of 39 vs. 0% in favor of patients without deletions (*p* = 0.000), which translated to a difference in OS of 49 vs. 0% (*p* = 0.001). This difference was preserved in the subgroup of Ph/−/ B-ALL, while there was no significant difference in the Ph/+/ B-ALL. The most frequently observed type of deletion (15 out of 19) was the Δ4–7. There is a strong negative prognostic impact of the *IKZF1* deletions at diagnosis in the observed population. *IKZF1* deletion testing through breakpoint-specific qPCR is a practical approach in diagnostic testing for this risk factor. *IKZF1* deletions may warrant treatment decisions and intensified treatment strategies to overcome the negative prognostic impact.

## 1. Introduction

**Figure 1:**
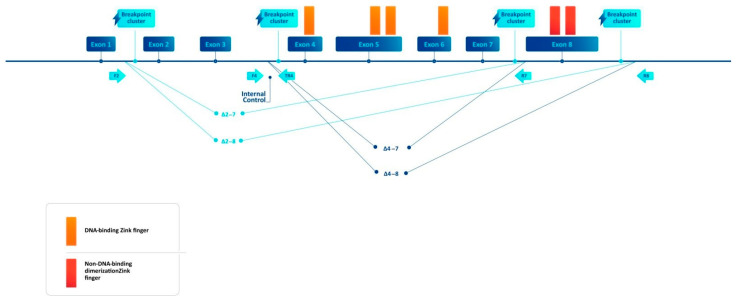
Structure of the *IKZF1* gene with the location of breakpoint clusters and primer locations.

The *Ikaros* zinc finger factor 1 (*IKZF1*) is a transcription factor with a well-known role in B- and T-cell development [1,2,3,4]. The *IKZF1* gene is located on chromosome 7p12.2 and includes eight exons, coding the DNA-binding transcription factor of the zinc finger family. The structure of *Ikaros* involves a DNA-binding domain with four zinc finger motifs and a dimerization domain with two zinc finger motifs (Figure 1). The physiological forms of *Ikaros*—Ik1 and Ik2—have four or three DNA-binding ZF motifs and have preserved DNA-binding activity. Isoforms resulting from intragenic deletions that miss two or more of the DNA-binding ZF motifs have reduced DNA-binding activity (Ik4, Ik5, Ik6, Ik7, Ik8) [5,6]. The importance of *IKZF1* in human pathology is demonstrated by reports assessing its significance among a range of conditions, such as infection, autoimmunity, solid tumors, myeloproliferative neoplasms, and lymphoid malignancies [7,8,9]. *IKZF1* deletions have also been observed in acute lymphoblastic leukemia/lymphoblastic lymphoma (ALL/LBL) [10,11,12,13,14,15,16]. There have been conflicting reports regarding the incidence and, particularly, the significance of *IKZF1* deletions in acute lymphoblastic leukemia. The incidence rates of *IKZF1* deletions in pediatric B-ALL cases are assessed by different groups as 28.6% [11], 16% [17], and 16% [18]. The incidence rates of *IKZF1* deletions in adult Ph/+/ B-ALL is the highest reported, but still with figures ranging between 63% [14], 91% [16], 41.3% [15], 43.4% [19], and 68.3% [20]. The incidence rates of *IKZF1* deletions in Ph/−/ B-ALL are reported as 26.6% [13], 13.6% [15], 24.6% [19], and 21.4% [20]. The incidence rate in T-ALL is usually the lowest reported at 3% [10], and 4% [21], but a group has found shorter isoforms related to deletion in all of 18 T-ALL samples [22]. The variability in terms of the prognostic significance is even greater. Pediatric B-ALL 5-year event-free survival (EFS) has been quoted at 25.2%, with a statistically significant impact of the *IKZF1* deletions [11]. Another large study quoted 40% cumulative incidence of relapse, which is significantly higher in patients with deletions. A DFCI (Dana-Farber Cancer Institute) team assessed the statistically significant impact on the 5-year EFS at 63% [18]. The Ph/+/ B-ALL 71% 2-year overall survival (OS) without statistically significant impact [14] was reported by an Italian team, while a UK team found no statistically significant impact on EFS and OS [15], and a Japanese team found statistically significant positive prognostic impact for dominant negative isoforms (DNI) on overall survival [20]. In Ph/−/ B-ALL a German group reports 46% 5-year OS for all types of *IKZF1* deletion with a trend to statistical significance, and 35% 5-year OS for loss of function (LOF) deletions with a significant impact [13], but a UK team found no statistically significant impact on EFS and OS [15]. In T-ALL, a French group reported 30% 5-year OS, which is statistically significant [10], and a Japanese team found a statistically significant positive impact on OS of DNI (dominant negative isoform) forms with a 3-year OS of 100% [20]. The different reports can be explained in part by different patient characteristics, different treatment modalities, different prevalences in the respective subgroups of ALL/LBL, potential ethnicity-related differences [23], and the different testing methods employed. The modalities involved for the testing included, among others, reverse transcriptase polymerase chain reaction (RT-PCR), multiplex ligation-dependent probe amplification (MLPA), sequencing techniques, and DNA–based techniques, such as breakpoint-specific PCR [13,14,15,16,19,24,25,26,27,28]. Historically, the RNA-based techniques were the first employed in the study of the *IKZF1* factor. They helped to identify the leading concepts of *IKZF1* aberrations such as the DNI, Ik4, Ik6, Ik8, and Ik10. The DNIs have reduced DNA-binding activity and have suppressed the DNA binding of physiological isoforms. The DNA-based techniques are built on the knowledge of the isoforms and are designed to seek specific types of deletions, with Δ4–7, producing the Ik6 isoform, being the most frequently observed [13,15]. Broader deletions which involve the dimerization domains or start codon are called loss of function deletions (Δ2–7, Δ2–8, Δ4–8) [13]. While correlation between the different methods for the frequent deletions has been demonstrated for the RT-PCR and qPCR [13], the sheer multitude of techniques employed suggests that each of the methods has advantages and disadvantages, and there is further need to define a pragmatic approach towards the optimal way to test for the *IKZF1* deletions.

## 2. Materials and Methods

### 2.1. Patients

Our study retrospectively analyzes 78 adult patients with ALL/LBL (age 16–77, median age 41), for whom diagnostic samples from bone marrow or peripheral blood, appropriate for DNA-PCR, were available. The patients were diagnosed between 2010 and 2022 in the Specialized Hospital for Active Treatment of Hematology Diseases (SHATHD). All the subjects had signed the applicable informed consent form permitting scientific research on their samples. All the subjects were treated according to the applicable Bulgarian standards of care for acute lymphoblastic leukemia (adapted from BFM, GMALL 07/2003, GRAAL, HyperCVAD).

### 2.2. Breakpoint-Specific PCR Design

We chose a breakpoint-specific multiplex PCR reaction design to test for the most frequent types of *IKZF1* intragenic deletions: Δ4–7, Δ2–7, Δ4–8, Δ2–8 (from now on referred to as *IKZF1* deletions). We used the primer3Plus tool https://www.primer3plus.com (accessed on 5 January 2024) and the publicly available genomic sequence of the *IKZF1* gene published in Genbank https://www.ncbi.nlm.nih.gov/genbank/ (accessed on 10 October 2023). Our primers flanked the breakpoint clusters with the highest frequency of breakpoints as previously described [29] in intron 1, intron 3, intron 7, and the breakpoint cluster region distally to exon 8 (Figure 1). The primer design was optimized to produce optimal-sized bands with the PCR kits utilized by our team, and to produce discernible differences between different deletions on gel electrophoresis. The primers used were F2—TTTGAAGCTTACAAGAAGAGAAACA, F4—TGGTCTTCTCCCAGCCCATA, R7—TCAACAGAGATCACAATAGATGGAAC, and R8—TCCTGCAACAATCTACCAGCA. The primers were combined in two wells for each reaction (F2, F4, and R7) and (F2, F4, and R8) for each sample. A reverse primer located in intron 3 (TR4—GCCACAACAGACATTTAACATGC) was added to both wells to amplify a continuous sequence from intron 3, which aimed to serve as an internal control. We used EURx^®^ (Gdansk, Poland) SG/ROX qPCR MasterMix (2×) under the following reaction conditions: an initial denaturation step at 95 °C for 10 min, followed by 35 cycles of denaturation at 94 °C for 15 s, annealing at 60 °C for 40 s, extension at 72 °C for 50 s, with a final extension at 72 for 10 min. We also utilized the primer pairs described previously by Caye et al. [24] under the above reaction conditions.

Electrophoresis of all PCR amplification products was performed on 1% agarose gels and run in a 1× TBE buffer EURx^®^ (Gdansk, Poland) using 6x Orange DNA Loading Dye Thermo Fisher Scientific^®^ (Waltham, MA, USA) and a GeneRuler Low Range DNA Ladder Thermo Fisher Scientific^®^ (Waltham, MA, USA) to demonstrate the different sized bands. 

### 2.3. Statistical Considerations

Data from the hospital information system of the Specialized hospital for active treatment of hematology disease (SHATHD) were used as a source of information for the studied patients. The statistical software package IBM SPSS Statistics 22 was used to conduct descriptive statistical and survival analyses. We used the Kaplan–Meier method to assess the effect of *IKZF1* deletions on event-free survival and overall survival of the population, and we conducted a Cox regression analysis of the effect of the conventional risk factors in ALL and *IKZF1* deleted status. A SAS on demand software package (https://welcome.oda.sas.com (accessed on 18 August 2024) was used to conduct cumulative risk of relapse analysis with death as a competing factor. Analyses were conducted for the impact of all the deletions on the entire population, in subgroups for Δ4–7 and LOF deletions on the entire population, and for all the deletions among Ph/+/ B-ALL and among Ph/−/ B-ALL. EFS and OS analyses were repeated with censoring for allogeneic hematopoietic stem cell transplant (HSCT), and separately for the frontline treatment protocol most frequently employed (adapted from the protocol of the German multicenter group for adult acute lymphoblastic leukemia—GMALL 07/2003 (NCT00198991)).

## 3. Results

Our population consisted of 78 patients with the median age at diagnosis of 41 (16–77). A total of 53 (68%) were B-ALL, and out of those, 40 (75%) were Ph/−/ B-ALL, 13 (25%) were Ph/+/ B-ALL, and 25 (32%) were T-ALL/LBL. Two of the T-ALL/LBL patients had less than 20% of bone marrow involvement at the time of diagnosis. The majority of the patients were treated according to the Bulgarian frontline standard of care treatment adapted from the protocol of GMALL07/2003–61 (78%). Allo-HSCT was conducted in 25 (32%). The complete remission rate in the entire population was 74%, and the 5-year EFS and 5-year OS for the entire population were, respectively, 31% and 39%. In our population, we discovered 19 (24%) patients positive for *IKZF1* deletions. The patient population characteristics are summarized in Table 1. Looking at the patient characteristics, we noted the larger share of standard risk patients per the GMALL07/2003 conventional risk factors at diagnosis [30] in the subgroup without deletions. We also noted that separately analyzed WBC count at diagnosis and bone marrow infiltration did not seem statistically significantly related to *IKZF1* deletion status. While MRD negativity after the first consolidation was less frequently achieved in the *IKZF1* deleted group, this was not statistically significant (Table 1).

**Figure 2:**
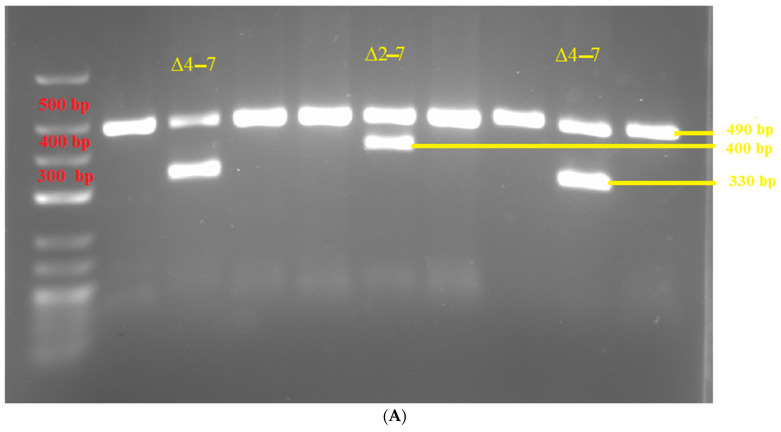

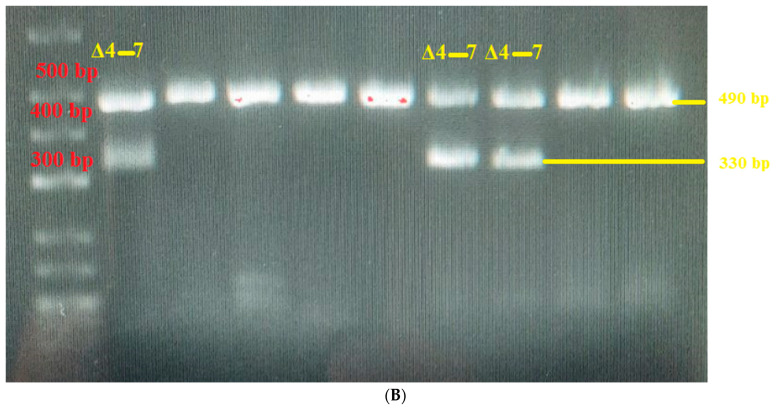
(**A**,**B**) Gel electrophoresis. (**A**) deletion of Δ2–7 and Δ4–7. (**B**) deletion of Δ4–7.

Out of the *IKZF1* deletions we found, the majority were Δ4–7 (15 out of 19). We found five Δ4–8 and one Δ2–7 (Figure 2). Two patients manifested deletions of both Δ4–7 and Δ4–8. All the deletions but one were in patients with B-ALL, and the single positive T-ALL/LBL patient manifested with Δ4–8 and did not have morphological bone marrow infiltration at the time of diagnosis. We did not observe deletion of Δ2–8 in our subset of patients. Per subsets, we observed 6 *Ikaros* deletions in Ph/+/ B-ALL (46%), 12 in Ph/−/ B-ALL (30%), and 1 in T-ALL/LBL (4%). Despite the lower CR rate in subjects with *IKZF1* deletions, Fisher’s exact test did not confirm significance of the relationship.

**Figure 3:**
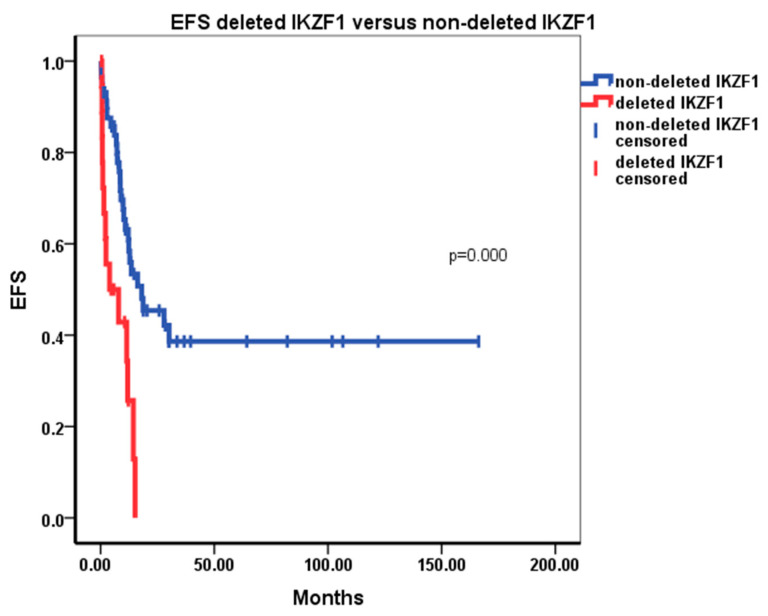
EFS for deleted *IKZF1* versus non-deleted *IKZF1* without censoring for HSCT.

**Figure 4:**
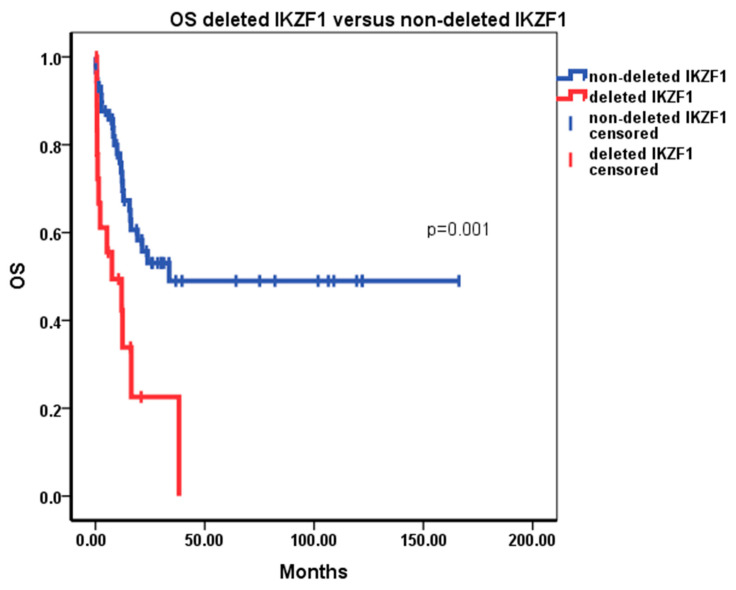
OS for deleted *IKZF1* versus non-deleted *IKZF1* without censoring for HSCT.

**Figure 5:**
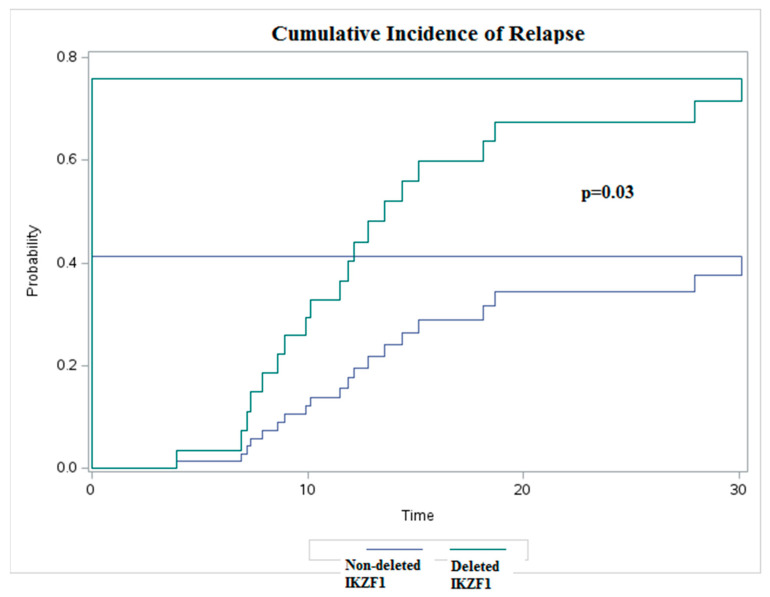
Cumulative incidence of relapse with death as competing factor deleted *IKZF1* vs. non-deleted *IKZF1*.

The 5-year EFS and the 5-year OS appeared significantly lower for the subjects with *IKZF1* deletions. The EFS was 0% at the fifth year for the patients with deletions versus 39% for the patients without deletions (*p =* 0.000) (Figure 3). The OS was 0% at the fifth year for the patients with *IKZF1* deletions versus 49% for patients without deletions (*p* = 0.001) (Figure 4). The analysis of the cumulative risk of relapse with death as the competing factor demonstrated statistically significant inferior outcomes in patients with *IKZF1* deletion (Figure 5).

The significant effect on EFS and OS observed in the population without censoring for allogeneic HSCT was preserved after censoring for allogeneic HSCT (*p* = 0.000 for EFS and *p* = 0.021 for OS). The Cox regression analysis of conventional prognostic factors and *IKZF1* deleted status for the entire population identified statistically significant (*p* < 0.05) hazard ratios (95% CI) for the risk groups per conventional prognostic factors per GMALL07/03HR 2.095 (1.169–3.756); *IKZF1* deleted status, HR 5.729 (1.881–17.442); failure to achieve remission after front-line induction, HR 129.9 (25.508–661.620); Ph/+/status, HR 9.43 (1.028–86.529); and age, HR 1.049 (1.015–1.084). In the Ph/+/ B-ALL subgroup, although the median EFS and OS were higher in the *IKZF1* non-deleted group—8 vs. 2 months and 15 versus 2 months, respectively—these differences were not statistically significant (*p* = 0.849, and *p* = 0.649, respectively). In the Ph/−/ B-ALL subgroup, *IKZF1* deletions continued to demonstrate statistically significant effect on the EFS (median 16 months versus 8 months, *p* = 0.005) and OS (median 24 months versus 8 months for patients with deletions, *p* = 0.044). The subgroup analyses from patients treated only with the adapted GMALL07/2003 protocol demonstrated median EFS of 12 months for patients with *IKZF1* deletions, 16 months for those without deletions (*p* = 0.015), and OS of 12 vs. 24 months (*p* = 0.024). The analyses of subjects with deletion of Δ4–7 confirmed its statistically significant prognostic impact on EFS and OS remained when the patients with LOF deletions were removed from the analyses group (*p* = 0.001 and 0.000, respectively). The analyses of EFS and OS of patients with LOF deletions versus those without deletions did not demonstrate a statistically significant effect on EFS and OS.

The characteristics of the *IKZF1* del positive patients are outlined in Table 2.

## 4. Discussion

The overall prevalence of *IKZF1* deletions found in our group falls within the broad ranges of earlier reports. Looking into the subgroup frequencies, we noted that in our group the prevalence of *Ikaros* deletions among Ph/+/ B-ALL patients is in the lower range of reported figures, while the prevalence in Ph/−/ B-ALL is slightly higher than the previously reported figures. While the differences cannot be ruled out to be ethnicity-related, they are relatively small and can also be due to statistical variation within a smaller sample size. To confirm a statistically significant 4% difference in incidence rate due to background, a sample of 1977 patients would be needed as per sample size test.

The single T-ALL/LBL patient who had a positive sample for *IKZF1* deletion from their bone marrow at diagnosis was suspected to have a minimum infiltration of the marrow of around 0.5% T-cell precursors at diagnosis per flow cytometry. While similar breakpoint qPCR designs have been demonstrated to be capable of detecting MRD at the level of 10^−4^ [24], the design of our protocol was not intended to achieve such levels of sensitivity. It should be noted that recent findings have proposed a role for constitutional *IKZF1* mutations in immunity, and have been related through immune deficiency with cases of T-ALL/LBL. While these *IKZF1* mutations are different than the deletions discussed in this article, it has also been demonstrated that pathogenesis mechanisms of the mutations are very similar to those of the deletions, causing dominant negative effects on the DNA-binding ability or causing loss of dimerization capability [31].

The patients in our subgroup did very poorly in terms of outcome. While our study was not powered to demonstrate statistically significant differences in CR rates between the groups, only 63.2% of the patients with *IKZF1* deletion achieved remission, which was a major contribution to the dismal outcomes of the group. We noted that the differences between reports on the EFS and OS come from populations which are treated on similar but different treatment protocols. We hypothesize that a more intensive treatment, and particularly a more intensive induction period, may be able to overcome in full or in part any negative impact on CR, EFS, and OS. There are ongoing studies aiming to explore such treatment intensifications [32,33,34].

Our work has optimized a relatively simple, cost-effective, and accessible method even for laboratories with limited resource to test for the most common types of *IKZF1* intragenic deletions. While the significance of these abnormalities is known in the indication of ALL, the method may be useful for analyses in other malignancies and in immunity, as the role of the *IKZF1* deletions needs to be explored further and incorporated into treatment decisions.

## Figures and Tables

**Table 1:** Patient characteristics.

Characteristics	Patients with *IKZF1* Deletion		Patients without *IKZF1* Deletion	
	*n* = 19	24%	*n* = 59	76%
Median age	44		40	
Male	12	63.2	34	57.6
Female	7	36.8	25	42.4
B	18	94.7	35	59.3
T	1	5.26	24	40.7
Ph/+/	6	31.6	7	11.9
*KMT2A* rearranged	2	10.5	3	5.08
t (1; 19)	0	0	5	8.47
B-ALL Hypoploidy	1	1.69	1	1.69
B-ALL Hyperploidy	0	0	2	3.38
Other cytogenetic abnormalities not listed above	5	26.31	4	6.78
Mean WBC count *p* = 0.703	48.5		42.7	
Mean bone marrow involvement *p* = 0.297	67.2		73.8	
Conventional risk groups as per the GMALL07/2003 initial risk classification				
VHR	6	31.6	7	11.9
HR	9	47.4	29	49.2
SR	4	21.1	23	39
CR rate	12	63.2	46	78
Allogeneic transplant rate	4	21.1	21	35.6
MRD negative after first consolidation *p* = 0.259	5	41.7	26	56.5

**Table 2:** Characteristics of the patients with *IKZF1* deletions.

Patient Number	Sex	Age	WHO Diagnosis	*IKZF1*del	Cytogenetics	Molecular Studies	CR	AlloHSCT
3	m	45	T-ALL/LBL	Δ4–8	normal karyotype	not conducted	not achieved	No
4	f	38	B-ALL NOS	Δ4–7	Trisomy 21 in 15% of the metaphasic plates	no abnormalities	achieved CR1	No
12	m	73	B-ALL NOS	Δ4–7 and Δ4–8	normal karyotype	no abnormalities	not achieved	No
13	m	35	B-ALL *KMT2A* rearranged	Δ4–7	t (4; 11)	MLL-AF4	achieved CR1	Yes
17	m	35	B-ALL t (9; 22)	Δ4–7	t (9; 22)	m BCR-ABL (p190)	not achieved	No
20	m	44	B-ALL NOS	Δ2–7	46, XX, t (3; 6) (q21; p23), −5, −7, add (12) (p13), +2 mar [9]/47, XYY [1]/46, XY [2]	no abnormalities	achieved CR1	No
23	m	41	B-ALL t (9; 22)	Δ4–7	t (9; 22) and near-tetraploid karyotype	m BCR-ABL (p190)	achieved CR1	No
31	m	45	B-ALL NOS	Δ4–7	46, XY, del (3) (q22q26–27) [8]/46, XY [8]	no abnormalities	achieved CR1	Yes
47	m	30	B-ALL t (9; 22)	Δ4–7	t (9; 22)	m-BCR-ABL (p190)	achieved CR1	Yes
48	f	47	B-ALL NOS	Δ4–7	del 13q	no abnormalities	achieved CR1	No
49	f	23	B-ALL NOS	Δ4–7	normal karyotype	no abnormalities	not achieved	No
55	m	16	B-ALL NOS	Δ4–8	no growth	no abnormalities	achieved CR1	Yes
75	m	50	B-ALL t (9; 22)	Δ4–8	t (9; 22)	M-BCR-ABL: p210	achieved CR1	No
77	f	55	B-ALL t (9; 22)	Δ4–7	t (9; 22)	m-BCR-ABL (p190)	achieved CR1	No
84	f	37	B-ALL NOS	Δ4–7	45, XX, −9 [7]/43–44, XX, −7 [1], −8 [5], −9, −13 [1], −22 [1], +mar [1] [cp 7].	no abnormalities	not achieved	No
85	f	71	B-ALL NOS	Δ4–7	normal karyotype	no abnormalities	achieved CR1	No
88	f	30	B-ALL *KMT2A* rearranged	Δ4–7 and Δ4–8	t (4; 11) (q21; q23)	MLL-AF4	achieved CR1	No
89	m	45	B-ALL t (9; 22)	Δ4–7	no growth	M-BCR-ABL p210	not achieved	No
101	m	50	B-ALL NOS	Δ4–7	normal karyotype	no abnormalities	not achieved	No

## Data Availability

The raw data supporting the conclusions of this article will be made available by the authors on request.

## References

[1] Georgopoulos K., Winandy S., Avitahl N. (1997). The role of the Ikaros gene in lymphocyte development and homeostasis. Annu. Rev. Immunol..

[2] Sridharan R., Smale S.T. (2007). Predominant interaction of both Ikaros and Helios with the NuRD complex in immature thymocytes. J. Biol. Chem..

[3] Georgopoulos K., Moore D.D., Derfler B. (1992). Ikaros, an early lymphoid-specific transcription factor and a putative mediator for T cell commitment. Science.

[4] Payne M.A. (2011). Zinc finger structure-function in Ikaros Marvin A Payne. World J. Biol. Chem..

[5] Molnár A., Georgopoulos K. (1994). The Ikaros gene encodes a family of functionally diverse zinc finger DNA-binding proteins. Mol. Cell Biol..

[6] Meyer C., Stadt U.Z., Escherich G., Hofmann J., Binato R., Barbosa T.C., Emerenciano M., Pombo-De-Oliveira M.S., Horstmann M., Marschalek R. (2013). Refinement of IKZF1 recombination hotspots in pediatric BCP-ALL patients. Am. J. Blood Res..

[7] Bose P., Verstovsek S. (2016). Prognosis of Primary Myelofibrosis in the Genomic Era. Clin. Lymphoma Myeloma Leuk..

[8] Kisiel J.B., Raimondo M., Taylor W.R., Yab T.C., Mahoney D.W., Sun Z., Middha S., Baheti S., Zou H., Smyrk T.C. (2015). New DNA Methylation Markers for Pancreatic Cancer: Discovery, Tissue Validation, and Pilot Testing in Pancreatic Juice. Clin. Cancer Res..

[9] Kuehn H.S., Nunes-Santos C.J., Rosenzweig S.D. (2021). Germline IKZF1 mutations and their impact on immunity: IKAROS-associated diseases and pathophysiology. Expert Rev. Clin. Immunol..

[10] Simonin M., Lhermitte L., Dourthe M.-E., Lengliné E., Graux C., Grardel N., Cayuela J.-M., Arnoux I., Gandemer V., Ifrah N. (2021). *IKZF1* alterations predict poor prognosis in adult and pediatric T-ALL. Blood.

[11] Mullighan C.G., Su X., Zhang J., Radtke I., Phillips L.A.A., Miller C.B., Ma J., Liu W., Cheng C., Schulman B.A. (2009). Deletion of IKZF1 and prognosis in acute lymphoblastic leukemia. N Engl. J. Med..

[12] Kastner P., Chan S. (2011). Role of Ikaros in T-cell acute lymphoblastic leukemia. World J. Biol. Chem..

[13] Kobitzsch B., Gökbuget N., Schwartz S., Reinhardt R., Brüggemann M., Viardot A., Wäsch R., Starck M., Thiel E., Hoelzer D. (2017). Loss-of-function but not dominant-negative intragenic IKZF1 deletions are associated with an adverse prognosis in adult BCR-ABL-negative acute lymphoblastic leukemia. Haematologica.

[14] Martinelli G., Iacobucci I., Storlazzi C.T., Vignetti M., Paoloni F., Cilloni D., Soverini S., Vitale A., Chiaretti S., Cimino G. (2009). IKZF1 (Ikaros) deletions in BCR-ABL1-positive acute lymphoblastic leukemia are associated with short disease-free survival and high rate of cumulative incidence of relapse: A GIMEMA AL WP report. J. Clin. Oncol..

[15] Mitchell R.J., Kirkwood A.A., Barretta E., Clifton-Hadley L., Lawrie E., Lee S., Leongamornlert D., Marks D.I., McMillan A.K., Menne T.F. (2021). IKZF1 alterations are not associated with outcome in 498 adults with B-precursor ALL enrolled in the UKALL14 trial. Blood Adv..

[16] Iacobucci I., Lonetti A., Messa F., Cilloni D., Arruga F., Ottaviani E., Paolini S., Papayannidis C., Piccaluga P.P., Giannoulia P. (2008). Expression of spliced oncogenic Ikaros isoforms in Philadelphia-positive acute lymphoblastic leukemia patients treated with tyrosine kinase inhibitors: Implications for a new mechanism of resistance. Blood.

[17] Van Der Veer A., Waanders E., Pieters R., Willemse M.E., Van Reijmersdal S.V., Russell L.J., Harrison C.J., Evans W.E., Van Der Velden V.H., Hoogerbrugge P.M. (2013). Independent prognostic value of BCR-ABL1-like signature and IKZF1 deletion, but not high CRLF2 expression, in children with B-cell precursor ALL. Blood.

[18] Vrooman L.M., Blonquist T.M., Harris M.H., Stevenson K.E., Place A.E., Hunt S.K., O’brien J.E., Asselin B.L., Athale U.H., Clavell L.A. (2018). Refining risk classification in childhood B acute lymphoblastic leukemia: Results of DFCI ALL Consortium Protocol 05-001. Blood Adv..

[19] Kimura H., Onozawa M., Yoshida S., Miyashita N., Yokoyama S., Matsukawa T., Hirabayashi S., Goto H., Endo T., Oguri S. (2023). Dominant-negative type of IKZF1 deletion showed a favorable prognosis in adult B-cell acute lymphoblastic leukemia. Ann. Hematol..

[20] Kimura H., Onozawa M., Yoshida S., Miyashita N., Takahashi S., Yokoyama S., Matsukawa T., Hirabayashi S., Fujisawa S., Ota S. (2022). Better Prognosis of Dominant-Negative Type of *IKZF1* Deletion in *BCR-ABL*-positive Adult B-Cell Acute Lymphoblastic Leukemia. Blood.

[21] Marçais A., Jeannet R., Hernandez L., Soulier J., Sigaux F., Chan S., Kastner P. (2010). Genetic inactivation of Ikaros is a rare event in human T-ALL. Leuk. Res..

[22] Sun L., Crotty M.L., Sensel M., Sather H., Navara C., Nachman J., Steinherz P.G., Gaynon P.S., Seibel N., Mao C. (1999). Expression of dominant-negative Ikaros isoforms in T-cell acute lymphoblastic leukemia. Clin. Cancer Res..

[23] de Smith A.J., Wahlster L., Jeon S., Kachuri L., Black S., Langie J., Cato L.D., Nakatsuka N., Chan T.-F., Xia G. (2024). A noncoding regulatory variant in IKZF1 increases acute lymphoblastic leukemia risk in Hispanic/Latino children. Cell Genom..

[24] Caye A., Beldjord K., Mass-Malo K., Drunat S., Soulier J., Gandemer V., Baruchel A., Bertrand Y., Cavé H., Clappier E. (2013). Breakpoint-specific multiplex polymerase chain reaction allows the detection of IKZF1 intragenic deletions and minimal residual disease monitoring in B-cell precursor acute lymphoblastic leukemia. Haematologica.

[25] Reyes-León A., Juárez-Velázquez R., Medrano-Hernández A., Cuenca-Roldán T., Salas-Labadía C., Navarrete-Meneses M.d.P., Rivera-Luna R., López-Hernández G., Paredes-Aguilera R., Pérez-Vera P. (2015). Expression of Ik6 and Ik8 Isoforms and Their Association with Relapse and Death in Mexican Children with Acute Lymphoblastic Leukemia. PLoS ONE.

[26] Rezayee F., Eisfeldt J., Skaftason A., Öfverholm I., Sayyab S., Syvänen A.C., Maqbool K., Lilljebjörn H., Johansson B., Olsson-Arvidsson L. (2023). Feasibility to use whole-genome sequencing as a sole diagnostic method to detect genomic aberrations in pediatric B-cell acute lymphoblastic leukemia. Front. Oncol..

[27] Brown L.M., Lonsdale A., Zhu A., Davidson N.M., Schmidt B., Hawkins A., Wallach E., Martin M., Mechinaud F.M., Khaw S.L. (2020). The application of RNA sequencing for the diagnosis and genomic classification of pediatric acute lymphoblastic leukemia. Blood Adv..

[28] Iacobucci I., Storlazzi C.T., Cilloni D., Lonetti A., Ottaviani E., Soverini S., Astolfi A., Chiaretti S., Vitale A., Messa F. (2009). Identification and molecular characterization of recurrent genomic deletions on 7p12 in the IKZF1 gene in a large cohort of BCR-ABL1-positive acute lymphoblastic leukemia patients: On behalf of Gruppo Italiano Malattie Ematologiche dell’Adulto Acute Leukemia Working Party (GIMEMA AL WP). Blood.

[29] Lopes B.A., Meyer C., Bouzada H., Külp M., Maciel A.L.T., Larghero P., Barbosa T.C., Poubel C.P., Barbieri C., Venn N.C. (2023). The recombinome of IKZF1 deletions in B-cell precursor ALL. Leukemia.

[30] Gökbuget N., Kneba M., Raff T., Trautmann H., Bartram C.-R., Arnold R., Fietkau R., Freund M., Ganser A., Ludwig W.-D. (2012). Adult patients with acute lymphoblastic leukemia and molecular failure display a poor prognosis and are candidates for stem cell transplantation and targeted therapies. Blood.

[31] Kuehn H.S., Boast B., Rosenzweig S.D. (2023). Inborn errors of human IKAROS: LOF and GOF variants associated with primary immunodeficiency. Clin. Exp. Immunol..

[32] Yeoh A.E.J., Lu Y., Ni Chin W.H., Chiew E.K.H., Lim E.H., Li Z., Kham S.K.Y., Chan Y.H., Abdullah W.A., Lin H.P. (2018). Intensifying Treatment of Childhood B-Lymphoblastic Leukemia With *IKZF1* Deletion Reduces Relapse and Improves Overall Survival: Results of Malaysia-Singapore ALL 2010 Study. J. Clin. Oncol..

[33] Pieters R., de Groot-Kruseman H., Fiocco M., Verwer F., Van Overveld M., Sonneveld E., van der Velden V., Beverloo H.B., Bierings M., Dors N. (2023). Improved Outcome for ALL by Prolonging Therapy for *IKZF1* Deletion and Decreasing Therapy for Other Risk Groups. J. Clin. Oncol..

[34] Tran T.H., Langlois S., Meloche C., Caron M., Saint-Onge P., Rouette A., Bataille A.R., Jimenez-Cortes C., Sontag T., Bittencourt H. (2022). Whole-transcriptome analysis in acute lymphoblastic leukemia: A report from the DFCI ALL Consortium Protocol 16-001. Blood Adv..

